# Visual Interpretation of Biomedical Time Series Using Parzen Window-Based Density-Amplitude Domain Transformation

**DOI:** 10.1371/journal.pone.0163569

**Published:** 2016-09-28

**Authors:** Selahaddin Batuhan Akben, Ahmet Alkan

**Affiliations:** 1 Department of Computer Technologies, Bahce Vocational School, Osmaniye Korkut Ata University, Osmaniye, Turkey; 2 Department of Electrical & Electronics Engineering, Faculty of Engineering and Architecture, Kahramanmaras Sutcu Imam University, Kahramanmaras, Turkey; Tianjin University, CHINA

## Abstract

This study proposes a new method suitable for the visual analysis of biomedical time series that is based on the examination of biomedical signals in the density-amplitude domain. Toward this goal, we employed two publicly available datasets. In the first stage of the study, density coefficients were computed separately by using the Parzen Windowing method for each class of raw attribute data. Then, differences between classes were determined visually by using density coefficients and their related amplitudes. Visual interpretation of the processed data gave more successful classification results compared with the raw data in the first stage. Next the density-amplitude representations of the raw data were classified using classifiers (SVM, KNN and Naïve Bayes). The raw data (time-amplitude) and their frequency-amplitude representation were also classified using the same classification methods. The statistical results showed that the proposed method based on the density-amplitude representation increases the classification success up to 55% compared with methods using the time-amplitude domain and up to 75% compared with methods based on the frequency-amplitude domain. Finally, we have highlighted several statistical analysis suggestions as a result of the density-amplitude representation.

## Introduction

**Fig 1:**
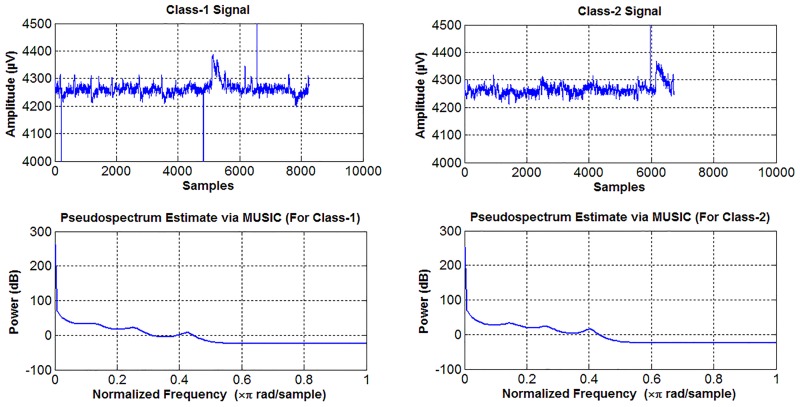
Display of a sample EEG signal represented in the time-amplitude and frequency-amplitude domains.

The diagnosis of related diseases is dependent on the visual differences between the signals obtained from healthy and unhealthy subjects. However, biomedical signals are generally obtained as time series in a time-amplitude domain, which can be visually complex [1]. Since, the visual analysis is difficult in a time-amplitude domain, feature extraction methods have been used as a pre-processing step. The most commonly used feature extraction method is the representation of the original signal in a frequency-amplitude domain [2–4]. Fourier transformation is a widely used and well-known method for frequency domain representation of raw signals. However, in a Fourier transformation, the frequency-amplitude representation is not always useful for visual analysis [5–7]. For example, Fig 1 shows a real EEG signal consisting of two different classes and their power spectral densities. As can be seen in Fig 1, it is difficult to distinguish the two signals in the time-amplitude and frequency-amplitude domains.

The main reason for this problem is that the frequencies and amplitudes of different signals may be the same or very similar over a prolonged period [8–10]. Therefore, different domain transformation methods based on a local examination are also used. For example, short-time Fourier and wavelet transformations are widely used for local examination. However, different signals may not always show local differences. Moreover local differences in the same class (healthy subjects or patients) may occur during different time periods [11–13]. Thus, in the literature, various feature extraction and classification methods have been used to overcome this problem [14, 15]. For example, other feature extraction methods such as an independent component analysis, principal component analysis or a modified version of Fourier analyses can be used. But the mathematical equations of these methods are more complex. Also, the representation of the raw signals in their created domains is difficult to understand except by signal processing experts. Therefore, it is very difficult for medical experts who need to make visual interpretations to use these methods [16]. As a result of these problems, an alternative method suitable to visual analysis and classification is needed.

The increase or decrease of the distance differences between samples is a common characteristic of digital biomedical time series (signals). For example, biomedical signals may sometimes contain samples that are far away from or close to each other. Since, the distance differences between samples do not occur throughout the entire signal, the visual detection of these differences is difficult [17]. Distance differences between samples in a specific time interval change the density of the signal in that interval. In this situation, the characteristic based on the distance between samples may be detected by determining the density difference. Therefore, density-amplitude domain representation of biomedical signals can be beneficial to visual interpretation.

In the first stage of this study, density coefficients of signal classes were obtained using the Parzen Windowing Method. Then, the class differences were visually examined in a density-amplitude representation. Upon visual examination, the characteristic differences of classes were visible in the density-amplitude domain representation. In addition, both the raw and transformed signals (all the signals represented in time-amplitude, frequency-amplitude and density-amplitude domains) could be used for the classification. The classification result demonstrated that the proposed method increases the classifier’s success. In a sense, the method proposed in this study is an improved version of the method proposed in our previous study [18]. In the previous study, time series were converted to density coefficients. However, visual interpretation of the data by using the coefficients was not possible. In this study, combined use of the amplitude values and corresponding density coefficients was proposed. Therefore, the method proposed in this study allows for visual interpretation of the data. However previous study doesn’t allow for visual interpretation of the data. Recently, complex network theory has been developed to characterize experimental univariate time series and multivariate time series [19–22].

## Materials and Methods

### Materials

EEG data represent a rather complex biomedical time series. Although many methods have been suggested, Fourier transformation-based frequency-amplitude domain representation is still widely used to process the EEG data. [23]. However, visual detection of differences between EEG signals in the time-amplitude and time-frequency domains without pre-processing methods is difficult. Therefore the EEG signals are suitable for testing the proposed method. In this study, the EEG Eye State biomedical dataset was used for a detailed analysis. This dataset consists of EEG records with eyes open or closed. Therefore, there are two classes in the dataset. Each attribute of the dataset includes a signal from one EEG channel (amplitude data). The dataset was obtained from one subject. The duration of the measurement was 117 seconds. The sampling rate was 128 Hz. For each channel, single-trial analysis was used to provide a systematic mapping between the brain activity and stimulus information space and test the proposed method for possible cases. Signals were filtered using a Low Pass filter with a cutoff that was set at the limit of the EEG gamma band. A notch filter at 50Hz was used to eliminate the line interference artifacts. In addition, linear-filter method was used to eliminate the other EEG artifacts. The dataset was taken from a publicly available UCI database (https://archive.ics.uci.edu/ml/machine-learning-databases/00264/) [24]. The characteristics of this dataset are shown in Table 1.

**Table 1:** Characteristics of the EEG Dataset Used in the Study.

Dataset	Number of Samples	Number of Attributes	Number of Classes
EEG Eye State	14980	14	2

In addition, another dataset (sEMG Hand Movement Dataset) taken from the UCI database (https://archive.ics.uci.edu/ml/machine-learning-databases/00313/) was used in the study to test and prove the classifier success of the proposed method [25]. There are six gestures of five volunteers in this data set. The aim is to distinguish the hand gestures for each volunteer. Characteristics of the sEMG data are as follows. The sampling rate was 500 Hz. The recording time was six seconds for each gesture. Signals were filtered using a Butterworth Band Pass filter with a low cutoff of 15Hz and high cutoff of 500Hz. A notch filter at 50Hz was used to eliminate the line interference artifacts. The general characteristics of the sEMG dataset can be seen in Table 2

**Table 2:** Characteristics of the EEG Dataset Used in the Study.

Dataset	Number of Samples	Number of Attributes	Number of Classes
EMG Hand Movement	3000	2500	6

### Methods

**Fig 2:**
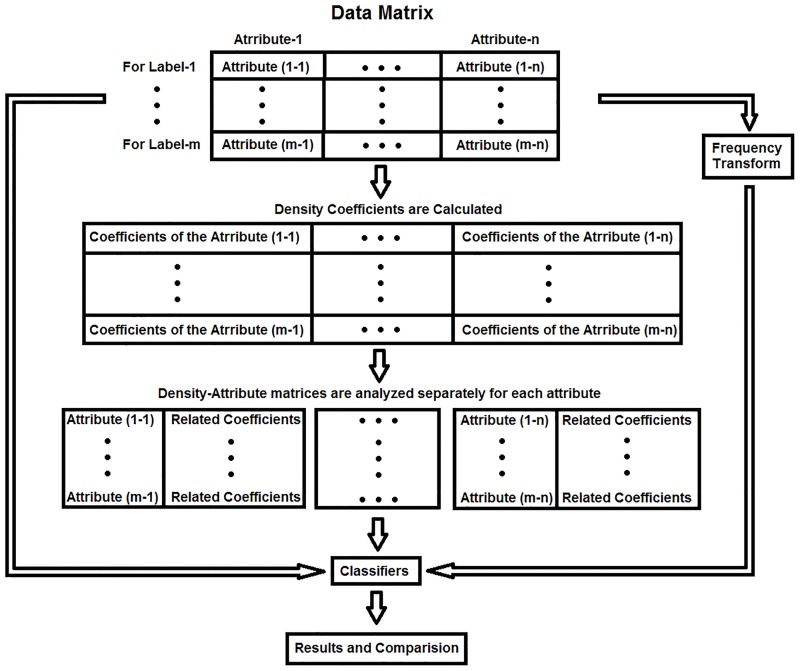
Flow diagram of the study (Attribute (m-n) means the class m data in the nth attribute).

**Fig 3:**
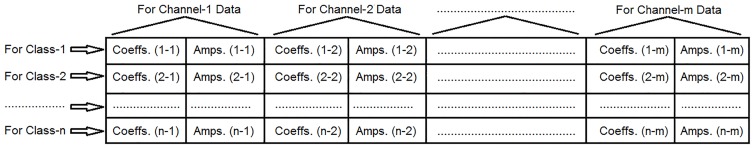
The matrix creation process used in the study (Amps. (n-m) means the class-n data of nth EEG channel signal).

In this study, the density coefficients were separately calculated for each class of each attribute (attributes are columns of the dataset). In other words, the density coefficients were calculated separately for each data class obtained from one EEG channel. Then, a new density-attribute matrix consisting of density coefficients and raw data (amplitude values that are the source of coefficients) was created by combining the old and new attributes in a single matrix. Next, the column pairs (two-column matrix parts that belong to each channel) consisting of the raw data and related coefficients were tested visually on two-axis graphs. The schematic block diagram of this study is shown in Fig 2. The obtained two-column matrices were tested by classification methods to determine the success statistically. The density-amplitude matrix creation process is shown in Fig 3.

It must be noted that the density coefficient calculation is made separately for each class, as shown in Fig 3. In other words, the density coefficient of a class element belonging to one channel is calculated considering only the other elements of the same class. In this study, the density coefficients were obtained by using the Parzen Windowing method. According to this method, the size and form of an *R* area (window) is fixed for the estimation of the density coefficients. To determine the density coefficient of an element, the *R* area is centered on this element, and the number of other elements in this area is calculated. In this case, the density value (coefficient) will be calculated by substituting the *φ*(u) function in Eq 2 to determine the elements to be included in the density calculation instead of the *k* function in Eq 1.

P(x)=knV (1)

In Eq 1, *x* is the sample (element), n is the total number of elements, and *V* is the volume (dimension) of the dataset.

$$\varphi \left(u\right)=\{\begin{array}{cc} 1,\; & \left|u_{j}\right|<\frac{1}{2},\;\;J=1,\ldots ,d\\ 0, & Otherwise \end{array}$$ (2)

As seen in Eq 2, the *φ*(*u*) value will be 1 if the |*u*_*j*_| element exists within the window, otherwise it will be 0. That is, any |*u*_*j*_| element will contribute to the density coefficient if it is within the window. When the *φ*(*u*) function in Eq 2 is substituted in the density function in Eq 1, the density coefficients of the elements will be calculated using Eq 3

$$P_{\varphi }\left(x\right)=\frac{1}{n}\Sigma_{i=1}^{i=n}\frac{1}{h^{d}}\varphi \left(\frac{x-x_{i}}{h}\right)$$ (3)

**Fig 4:**
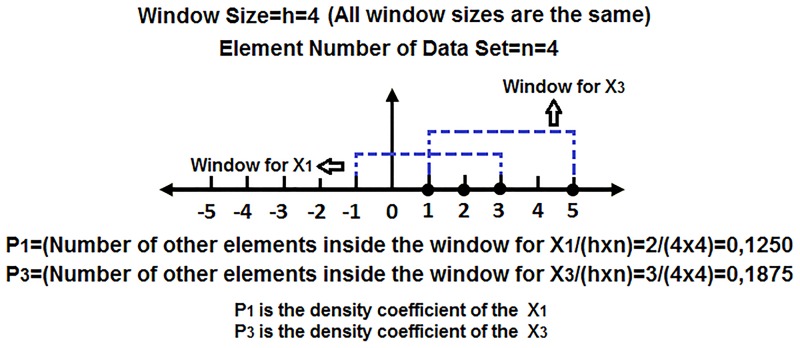
Calculation of the density coefficients for two elements in a sample dataset.

Where d is the dimension of the dataset [26–28]. Eq 3 means that the density coefficient of an element at the center of the window is proportional with the total number of the other elements within the window. Fig 4 shows a sample calculation of the density coefficients for *X*_*1*_ and *X*_*3*_ elements of dataset X, *X = [X*_*1*_, *X*_*2*_, *X*_*3*_, *X*_*4*_*] = [(1*,*0) (2*,*0) (3*,*0)(5*,*0)]*.

The density coefficients of the sample dataset in Fig 4 are 0.1250 for *X*_*1*_, 0.1250 for *X*_*2*_, 0.1875 for *X*_*3*_ and 0.625 for *X*_*4*_. As seen in Fig 4, for each coefficient calculation, at least one element contributes to the calculation of the coefficient. This is achieved because of the selection of the window width as two times the maximum distance between the elements. This means that the window width must be at least two times the maximum distance between the elements in order to provide density coefficient for all of the elements. However, another issue that must be taken into consideration is that the coefficients must be as different as possible. If the window size is more than required (more than two times), many coefficients will be same as each other, and this will decrease the classifier accuracy [29, 30]. In this case, the more distant element gives the lowest density coefficient in the dataset. The coefficient of an element located at the center of the densest area in the dataset will be the biggest. As seen in Fig 4, the largest distance between the elements is 2 (the distance between the elements located at 3 and 5 on the horizontal axis), and the window width is two times this value. Therefore, the density coefficient of *X*_*3*_ located in the densest area was the highest (0.1875), and the density coefficient of *X*_*4*_ located in the rarest area was found to be the lowest (0.6250). Furthermore, it was possible to assign density coefficients to all the elements, and the density coefficients were calculated as different from each other as possible.

**Fig 5:**
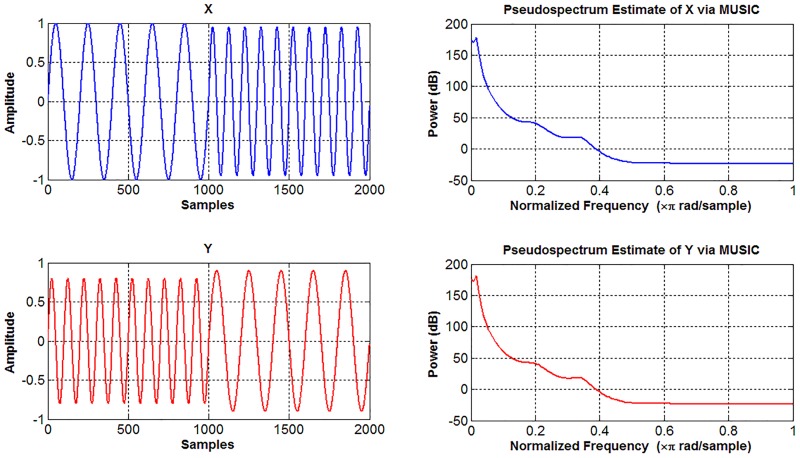
Display of two different signals represented in the time-amplitude and frequency-amplitude domains.

Since the objective of this study is to create a model that will allow the visual differentiation of signals that are difficult to differentiate in time-amplitude and frequency-amplitude domains, we used the example signals shown in Fig 5.

In Fig 5, two different signals and their frequency spectrums are shown. Both of the signals consist of two sinusoidal components of 5 Hz and 10 Hz. The occurrence times of the components of the signals are different. The amplitudes of the signal components having the same frequency are different from each other by 10%. This amplitude difference can be identified if the example signals are examined carefully. However, such small differences are continuous in biomedical signals, and thus visual detection of such differences would be rather difficult. The frequency spectra of the signals are almost the due to the same frequency content, a common issue in biomedical signals. Very detailed examinations or some preliminary procedures are required to analyze such small differences, particularly on EEG records consisting of thousands of samples. These types of signals cause failure in classification [31].

**Fig 6:**
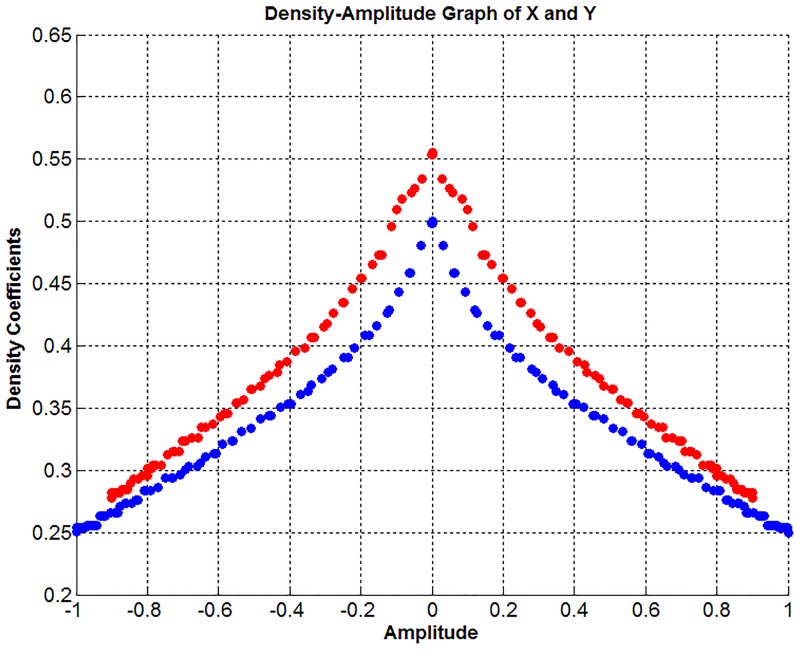
Density-amplitude domain representation of 3rd channel data (Blue: class-1 data, Red: class-2 data).

Considering the signal-X in Fig 5, the first half is 5 Hz and the second half is 10 Hz sine waves. This situation is reversed in the signal-Y, which causes dense locations of the signals that are different. As an average calculation, the maximum amplitudes of the signal-Y components are 10% less than those of signal-X. In this case, the difference between the example signals in Fig 5 is clearly revealed by the relation between their amplitudes and related coefficients. When the proposed method is used for the sample signals shown in Fig 5, the density-amplitude domain is as shown in Fig 6.

Fig 6 shows that the signals are no longer similar, and the difference between them can be visually detected easily. As also seen in Fig 6, the differences in the densities of the amplitudes near zero are greater because the density coefficients of amplitudes with lower values increased as the signal-Y amplitudes increased closer to zero. According to another definition, the density coefficient increase is inversely proportional to the scattering of the elements of the signal. In summary, the proposed method will be useful in visually differentiating the signals that are difficult to differentiate in time-amplitude and frequency-amplitude domains.

In addition, the well-known ROC analysis and Cross Validation (CV) methods were used to obtain more reliable results. According to the conventions of the CV method, in each cycle 90% of the data was used as a training set and the remaining 10% was used as a test set. Using the ROC method, the accuracy, sensitivity, and specificity rates can be computed by:

$$\text{Accuracy}=\frac{\text{TP}+\text{TN}}{\text{P}+\text{N}}$$ (4)

$$\text{Sensitivity}=\frac{\text{TP}}{\text{P}}$$ (5)

$$\text{Specificity}=\frac{\text{TN}}{\text{N}}$$ (6)

Where TP is the number of correctly classified disorders (Disorders correctly classified as disorder), FP is the number of falsely classified disorders (Healthy ones incorrectly classified as disorder), TN is the number of correctly classified healthy subjects (Healthy ones correctly classified as healthy), and FN is the number of falsely classified healthy subjects (Disorders incorrectly classified as healthy). Also, P is the number of disorders, and N is the number of healthy subjects [32].

## Results and Discussion

**Fig 7:**
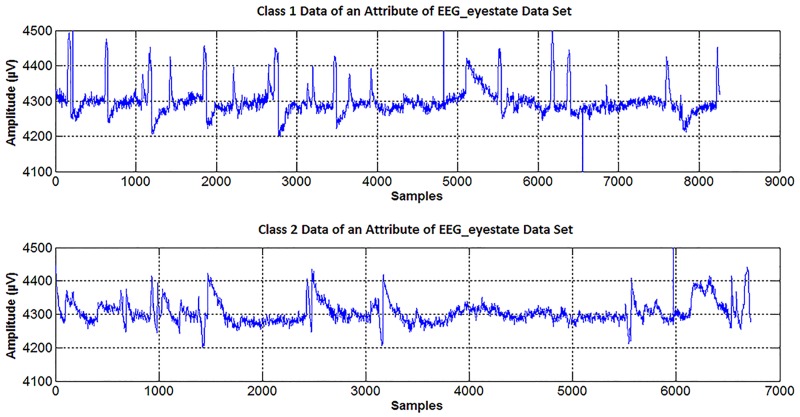
The time-amplitude representations of classes belonging to the same attribute. This attribute is also used in this study.

**Fig 8:**
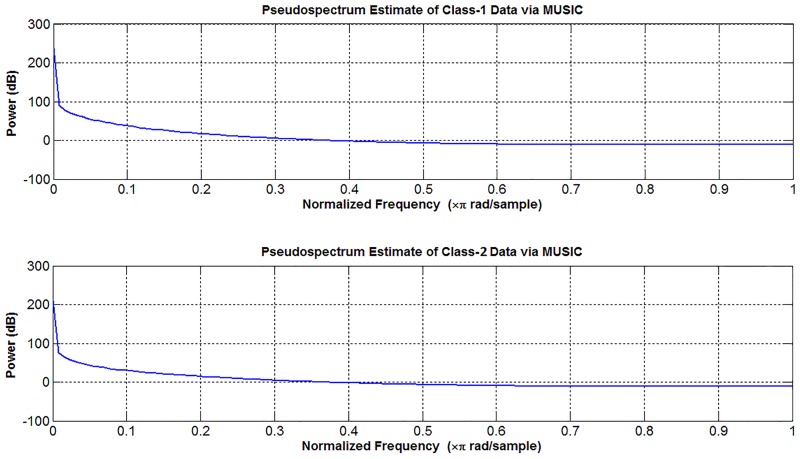
The frequency-amplitude representations of classes belonging to the same attribute. This attribute is also used in Fig 7.

The EEG dataset used in this study has periods recorded when eyes are opened or closed. The aim is to differentiate/classify the two classes consisting of closed eye and opened eye periods. In the first stage, the visual interpretation advantage of the proposed method will be shown on a randomly selected attribute of the EEG data matrix. Then, the results of the proposed method will be applied to the classifiers to evaluate the classification accuracies. The time-amplitude and frequency-amplitude representations of one attribute (third column of the dataset) are shown in Figs 7 and 8, respectively.

The peaks shown in Fig 7 may be due to eye movement or other causes [33]. For this reason, it is difficult to determine whether the eye is open or closed using the graphs in Fig 7. In Fig 8, it is impossible to determine the change in eye status (open/closed) without performing some preliminary procedures. However, the difference between the eye situations can be revealed by using the density-amplitude relationship.

**Fig 9:**
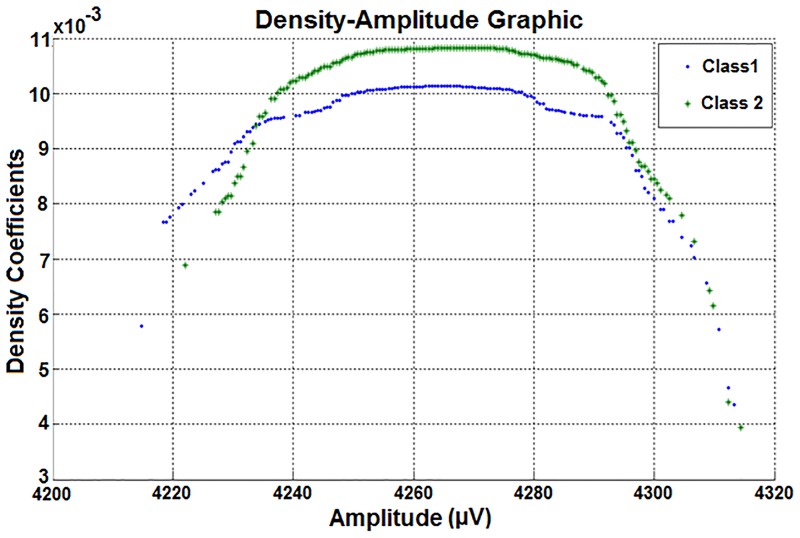
The density-amplitude representations of classes belonging to the same attribute. This attribute is also used in Figs 7 and 8.

Therefore, the two column density-amplitude matrix of the attribute used in Figs 7 and 8 was obtained. Then the density-amplitude graph was created by using the two column matrix values shown in Fig 9.

As shown in the distributions in Fig 9, there is a visible density difference between the class amplitude values for all of the points except the outliers. Thus, it can be seen that the data density is lower when the eyes are closed.

Furthermore, the biggest density-difference between the classes is across the average of the amplitude values of the dataset. The class differences seen in the Density-Amplitude domain can’t be detected in the Time-Amplitude or Frequency-Amplitude domains of the same data.

When Fig 9 is examined from another perspective, the density difference occurs between 4235 μV and 4290 μV. That is, the classes are different between these amplitude values. These values are very close to the standard deviation range of the density-amplitude distributions. If the amplitudes within the standard deviation of the density-amplitude distribution are filtered, the classifier success will be further improved. Moreover, the statistical values (including standard deviation, mean value, etc.) can be examined visually. The classifier success is also expected to be very high since the classes can be differentiated visually. In the second stage of this study, the attributes (represented in Time-Amplitude, Frequency-Amplitude and Density-Amplitude domains) were subjected to classifiers to test the ability to distinguish between domains. The test results are shown in Table 3. 14 different channel data obtained from 1 subject were considered in Table 3. Also the number of Negatives (N) is 8257 and the number of Positives (P) is 6723 for each classification process.

**Table 3:** Classification Success Rates of EEG Datasets.

Classifiers		Time-Amplitude	Frequency-Amplitude	Density-Amplitude
**Accuracy Rate**	**KNN**	56.81%	60.04%	98.75%
	**Naive-Bayes**	68.91%	53.23%	91.62%
	**SVM**	57.56%	49.67%	9376%
**Sensitivity Rate**	**KNN**	60.18%	63.34%	99.36%
	**Naive-Bayes**	72.34%	56.72%	93.57%
	**SVM**	62.49%	51.68%	94.76%
**Specificity Rate**	**KNN**	53.44%	56.74%	98.14%
	**Naive-Bayes**	65.48%	49.74%	89.67%
	**SVM**	52.63%	47.66%	92.76%

The K parameter providing the highest accuracy rate was used in the KNN classifier. In addition, the Euclidean distance was used in the KNN classifier. In the SVM classifier, a linear kernel was used and the margin providing highest accuracy rate was selected (i.e., margin was selected so as to make training error small). The Maximum Likelihood Estimator (MLE) was used to select parameters in the Naive Bayes classifier. As seen in the results given in Table 3, the classification success of the density-amplitude matrices was the highest, supporting the visual differentiation ability. That is, the classifier success can be increased by representing the raw data in the density-amplitude domain.

**Fig 10:**
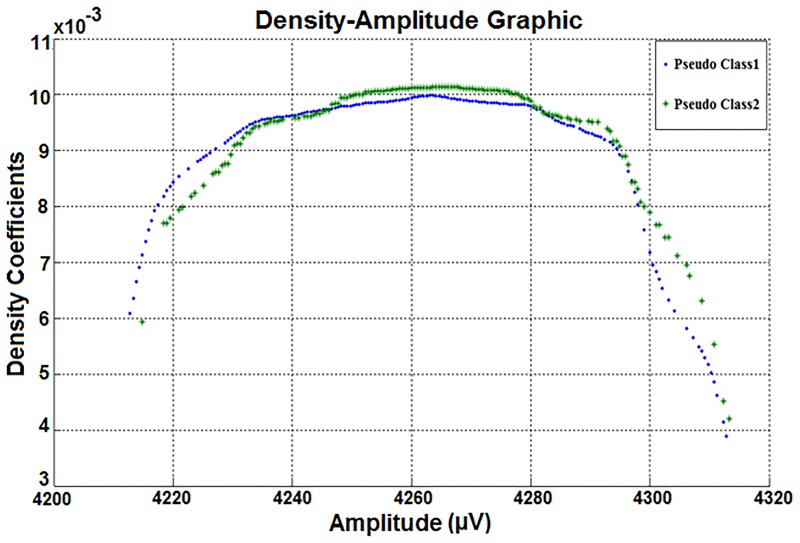
The density-amplitude graph of the 3rd attribute of the dataset (Class-1 is divided to get two fake (pseudo) classes. Both fake classes are related to the ‘eyes open’ status).

In this study, the attribute used in Fig 9 was tested to show that the distinguishing ability of the proposed method is not incidental. However, artificial classes were created by changing known class labels in this test stage. In the artificial class creation stage, the first and second halves of Class-1 were recognized as different classes. Then Class-1 was classified assumed as an attribute data. The Density-Amplitude graph of this artificial classified data is shown in Fig 10.

As seen in Fig 10, the class difference is not visible. Thus the proposed method is not incidental and reveals only the class differences.

Finally, the proposed method was tested using a different data set to demonstrate the success of the proposed method in a different biomedical time series. In this second classification stage, the EMG data set was used. The classifier results are shown in Table 4. Six different gestures requested 30 times from each of the 5 subjects were considered in Table 4. Also the number of Negatives (N) and the number of Positives (P) are 3000 for each classification process.

**Table 4:** Classification Success Rates of sEMG Datasets.

Classifiers		Time-Amplitude	Frequency-Amplitude	Density-Amplitude
**Accuracy Rate**	**KNN**	51.16%	54.21%	81.13%
	**Naive-Bayes**	54.27%	60.23%	85.39%
	**SVM**	53.56%	61.67%	86.56%
**Sensitivity Rate**	**KNN**	52.18%	55.56%	82.21%
	**Naive-Bayes**	55.23%	61.46%	86.78%
	**SVM**	54.63%	62.63%	87.29%
**Specificity Rate**	**KNN**	50.14%	52.86%	80.05%
	**Naive-Bayes**	53.31%	59.00%	84.00%
	**SVM**	52.49%	60.71%	85.82%

The K parameter providing the highest accuracy rate was used in the KNN classifier. In addition, the Euclidean distance was used in the KNN classifier. In the SVM classifier, a linear kernel was used and the margin providing highest accuracy rate was selected (i.e., margin was selected so as to make training error small). The Maximum Likelihood Estimator (MLE) was used to select parameters in the Naive Bayes classifier. As shown in Table 4, the proposed method increased the classifier success again.

In summary, the findings demonstrate the superiority of the proposed method over other methods in biomedical time series analyses. Its strength is related to biological induced convergence/divergence between the signal samples of biomedical time series. The proposed method converts convergence/divergence to density coefficients and then associates the amplitudes with related coefficients. Thus it offers a novel advantageous solution.

## Conclusion

In this study, 2 biomedical time series that are complex in the time-amplitude and frequency-amplitude domains were analyzed in the density-amplitude domain. The results demonstrated that the proposed method made possible the visual interpretation of characteristically complex biomedical time series. For example, visual inspection of amplitude differences between two different datasets, standard deviation differences and mean value differences were easily differentiated. As a result of these analyses, some diseases can be visually diagnosed in the density-amplitude domain, and biomedical time series can be classified with greater accuracy using the proposed method. While the proposed method uses only four basic arithmetic operations (+, -, × and ÷), the other methods use complex mathematical formulas. Therefore, the proposed method will be faster and clearer for medical experts. In addition, this easy calculation can permit an online evaluation while the signal is being recorded.

This method is especially useful for digital biomedical time series with sample convergence/divergence characteristics. Thus, the proposed method may also be more successful for local analysis. E.g., analysis of similar signal changes of two signals that may occur in different time periods will be examined to determine the difference between them in relation to time. If so, the use of the proposed method for local analysis may also be an alternative for STFT and wavelet transformations.

However, it must be noted that the proposed method may give erroneous results if the convergence/divergence of samples are not related to biological origin. This may appear as a disadvantage; however, this problem is true of all of the other methods, as well. Therefore, this is not a unique drawback of the proposed method. In other words, these sample types (samples are not biological originated) known as the outliers are problem for all methods. If so, this disadvantage can be avoided by filtering (outlier filtering) the samples that are not biological originated.

In conclusion, the obtained results will help expert medical doctors interpret data and will contribute to the visual and mathematical classification of biomedical time series. It provides an alternative to the frequency-amplitude based analysis of biomedical time series.
